# Phosphonated Iminodisuccinates—A Calcite Scale Inhibitor with Excellent Biodegradability

**DOI:** 10.1021/acsomega.2c06605

**Published:** 2022-12-28

**Authors:** Sumit Ganguly, Simen Tungesvik, Malcolm A. Kelland

**Affiliations:** Department of Mathematics and Natural Science, Faculty of Science and Technology, University of Stavanger, N-4036 Stavanger, Norway

## Abstract

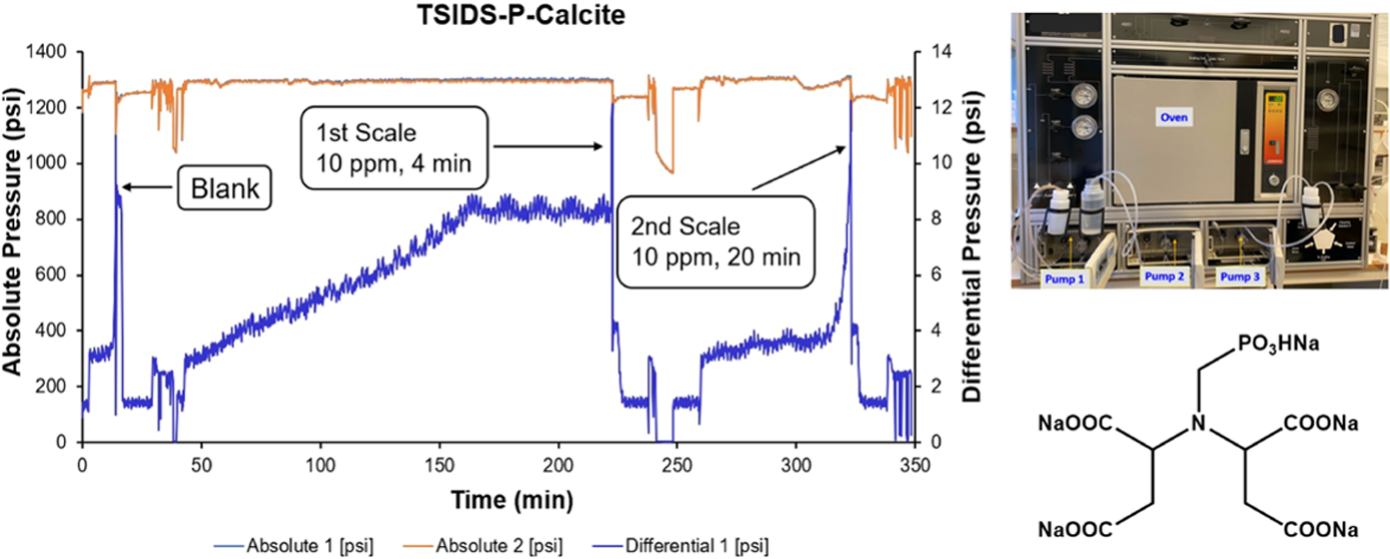

Scale inhibitors are an extremely important chemical in upstream oil and gas field operations and water treatment industries. These inhibitors prevent nucleation and/or crystal growth of scales such as calcite and barite. This keeps the pipes and other equipment and surfaces free from deposits, allowing the maximum flow of aqueous fluids. However, many classes of scale inhibitors are poorly biodegraded, especially in seawater, making them unacceptable in regions with strict environmental regulations. Tetrasodium iminodisuccinate (TSIDS) is a biodegradable, industrial-scale dissolver that we imagined could have potential as a scale inhibitor, given the correct derivatization. We first synthesized phosphonated derivatives of TSIDS (TSIDS-P) and the homologue phosphonate made from ethylenediamine disuccinate (TSEDAS-P). In particular, TSIDS-P was shown to be a good calcite scale inhibitor with good calcium compatibility but also exhibited over 70% biodegradation (BOD28) in the OECD 306 seawater test. This should make TSIDS-P a readily biodegradable scale inhibitor of great interest to the petroleum and water treatment industries.

## 1 Introduction

In the petroleum industry, deposition of insoluble mineral salts can occur in production wells, flow lines, and processing equipment from supersaturated produced aqueous fluids. These salt depositions are known as scale and are a major problem to the oil and gas industry during the upstream production phase.^1,2^ Inorganic scales can start forming on any surface and the layer can grow continuously in thickness if left untreated. If this occurs in the near well area, this can result in formation damage and loss of hydrocarbon production.^3,4^ The most typical oilfield scales encountered are calcium carbonate (calcite) and sulfate salts of calcium (gypsum), barium (barite), and strontium (celestite).^5^ Barite and calcite scales usually are noted to create quite extreme scaling scenarios inside production wells, and it is thus crucial for the oil industry to tackle this problem quickly and effectively so that production can run smoothly.

To prevent scale deposition, different polymeric or nonpolymeric chemicals known as scale inhibitors are used by oil companies. Scale inhibitors are water-soluble compounds, and they can be polymeric, containing mainly carboxylate and/or sulfonate groups or smaller nonpolymeric molecules with phosphonate groups.^6^ Some classical oilfield scale inhibitors being used worldwide are poly(acrylic acid) (PAA), poly(vinyl sulfonate) (PVS), copolymers of acrylic acid/vinyl sulfonate (AA/VS) or maleic acid/acrylic acid (MA/AA), and phosphonated amines like aminotris(methylenephosphonate) (ATMP), and diethylenetriamine penta(methylenephosphonate) (DTPMP).^7,8^

Phosphonated scale inhibitors are particularly good for downhole squeeze treatments, as they adsorb well to reservoir rock and can be easily detected in the produced water.^9−11^ Despite good performance, the major drawback of many phosphonate-based scale inhibitors is their lack of good biodegradability, which limits their use in areas where strict environmental regulations are being followed, such as offshore Norway. At the same time, many of these classic phosphonate scale inhibitors show poor tolerance toward high concentrations of calcium ions, resulting in precipitation and deposition as insoluble Ca^2+^–SI complexes.^12^

Therefore, there is a need for scale inhibitors that offer both good biodegradability (green inhibitors) and Ca-compatibility, along with appreciable inhibition performance.^13−15^ Over the past few years, our research group has explored various classes of phosphonate-based scale inhibitors with a view to meeting this goal. Several of such scale inhibitors developed in our lab exhibit excellent to moderate performance toward calcite and barite scale and also show good calcium tolerance.^16−22^ Here, we explore methylene-phosphonated derivatives of mono- and diaminopolycarboxylates, their calcite and barite scale inhibition performance, thermal stability, calcium compatibility, and seawater biodegradability.^23,24^ We were particularly inspired by the environmental profile of one aminopolycarboxylate, tetrasodium iminodisuccinate (TSIDS), which is reported to be readily biodegradable.^17^ We thought this would make an excellent starting point for phosphonate derivatives. Phosphonates of trisodium-ethylenediamine-*N*,*N*′-disuccinate are also reported. A different phosphonated derivative of TSIDS was reported while this work was ongoing.^18^

## 2 Experimental Section

### 2.1 Materials and Characterization

Tetrasodium iminodisuccinate was obtained from LANXESS (Commercial name Baypure CX100) as a 34% (w/w) aqueous solution. The ethylenediamine-*N*,*N*′-disuccinic acid trisodium salt solution (35% w/w in H_2_O) was purchased from Sigma-Aldrich. All other chemicals such as phosphorus acid (amorphous powder, 99%), formaldehyde (37% w/w aqueous solution), and HCl (AnalaR NORMAPUR, 37%) were received, respectively, from Aldrich, Alfa Aesar, and VWR Chemicals. All of the purchased chemicals were used without any further purification.

**Figure 1:**
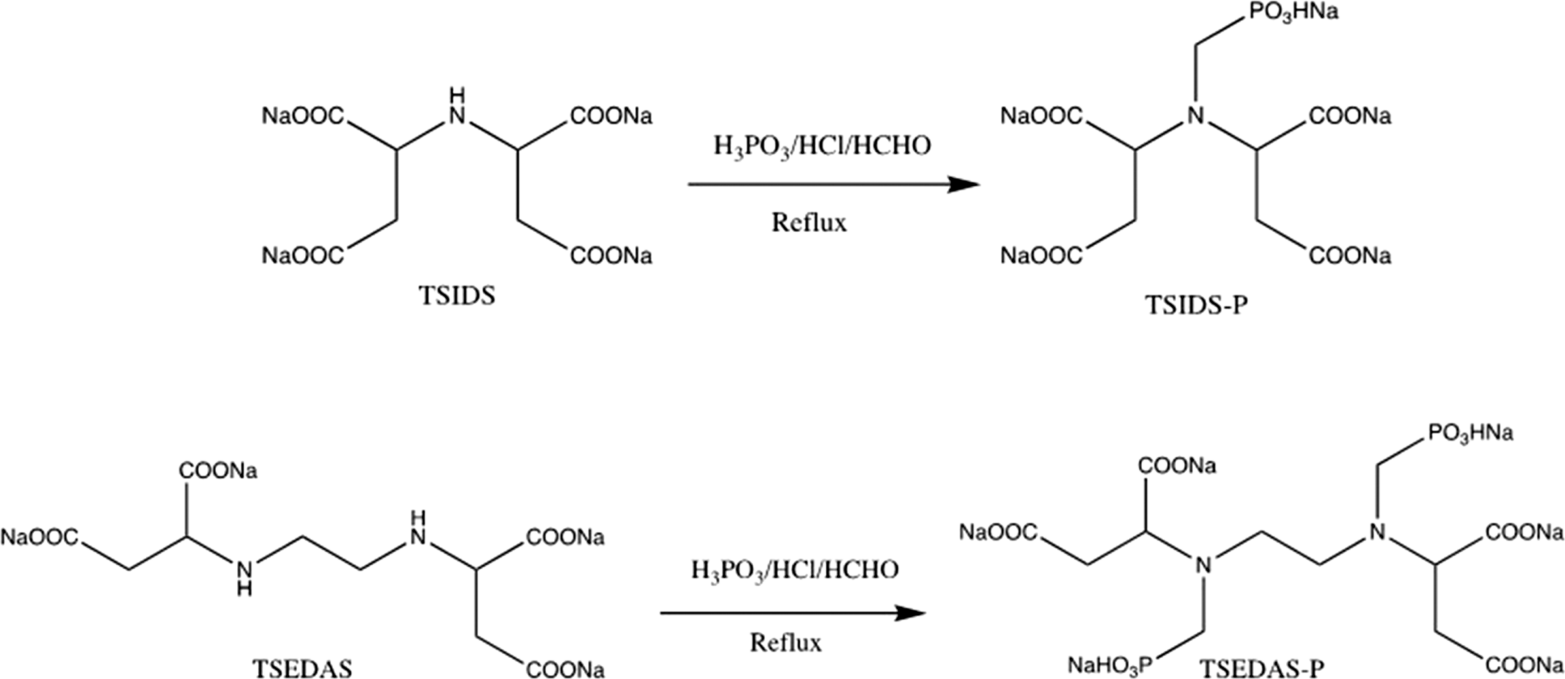
Synthesis of phosphonated aminosuccinate derivatives.

The syntheses carried out in this report are summarized in Figure 1. They use the well-known Moderitzi–Irani reaction for the methylene phosphonation of amines.^20^ Synthesized products were further structurally characterized by ^1^H and ^31^P NMR spectroscopy in a deuterium oxide (D_2_O) NMR solvent using a 400 MHz Bruker NMR spectrometer.

### 2.2 Synthesis of PhosphonatedTetrasodium-Iminodisuccinate (TSIDS-P)

Tetrasodium iminodisuccinate salt (2.01 g) (34 wt %, 5.96 mmol) along with 12 mL of distilled water were placed in a two-necked 100 mL round-bottomed flask, and to it was added phosphorous acid (0.59 g, 7.12 mmol) dissolved in 2–3 mL of distilled water. Subsequently, aqueous HCl (37 wt %, 3.53 g, 35.8 mmol) was added dropwise to the flask. The reaction flask, equipped with a reflux condenser and a nitrogen balloon, was heated stepwise at an oil bath to attain approx. 65 °C temperature. Afterward, an aqueous HCHO solution (37 wt %, 0.726 g, 8.94 mmol) was added via a syringe under the protection of nitrogen, and the reaction flask was set to 115 °C under reflux for 24 h. Upon completion, the flask was cooled to room temperature before adding the 20 wt % NaOH solution to neutralize the system to a pH of ≈ 6–7. The solution was evaporated to dryness, and the dry solid mass was collected for further tests and characterization. ^1^H NMR (D_2_O, 400 MHz) δ ppm: 6.06 (s, 1H), 3.94 (m, 1H, CH), 3.87 (m, 1H, CH), 3.29 (d, 2H, N–CH_2_–P−), 2.85 (m, 2H, CH_2_), 2.75 (m, 2H, CH_2_). ^31^P NMR (D_2_O, 162 MHz) δ ppm: 6.29 (t).

### 2.3 Synthesis of Phosphonated Trisodium-Ethylenediamine-*N*,*N*′-Disuccinate

**(TSEDAS-P):** Ethylenediamine disuccinate trisodium salt (1.0 g) (35 wt %, 2.79 mmol) and 8–9 mL of distilled water were placed in a two-necked 100 mL round-bottomed flask, followed by the addition of phosphorous acid (0.69 g, 8.37 mmol) dissolved in 2–3 mL of water. Afterward, HCl (37 wt %, 1.92 g, 19.53 mmol) was added dropwise to the reaction flask, and the flask was attached with a reflux condenser and a nitrogen balloon and was heated gradually in an oil bath up to approx. 65 °C temperature. An aqueous HCHO solution (37 wt %, 0.68 g, 8.37 mmol) was added to the solution via a syringe under the protection of nitrogen before the reaction temperature was raised to 115 °C, and the solution was left to reflux at that temperature for 24 h. Afterward, the reaction was cooled to room temperature, neutralized with aq. NaOH (30 wt %), and evaporated to dryness, and the dry solid mass was collected. ^1^H NMR (D_2_O, 400 MHz) δ ppm: 6.07 (s, 2H), 4.15 (m, 1H, CH), 4.06 (m, 1H, CH), 3.69 (m, 4H, N–CH_2_–P−), 3.15 (m, 3H, CH_2_), 2.92 (m, 5H, CH_2_). ^31^P NMR (D_2_O, 162 MHz) δ ppm: 8.93 (t), 8.32 (t).

### 2.4 High-Pressure Dynamic Tube-Blocking Test Protocol

**Figure 2:**
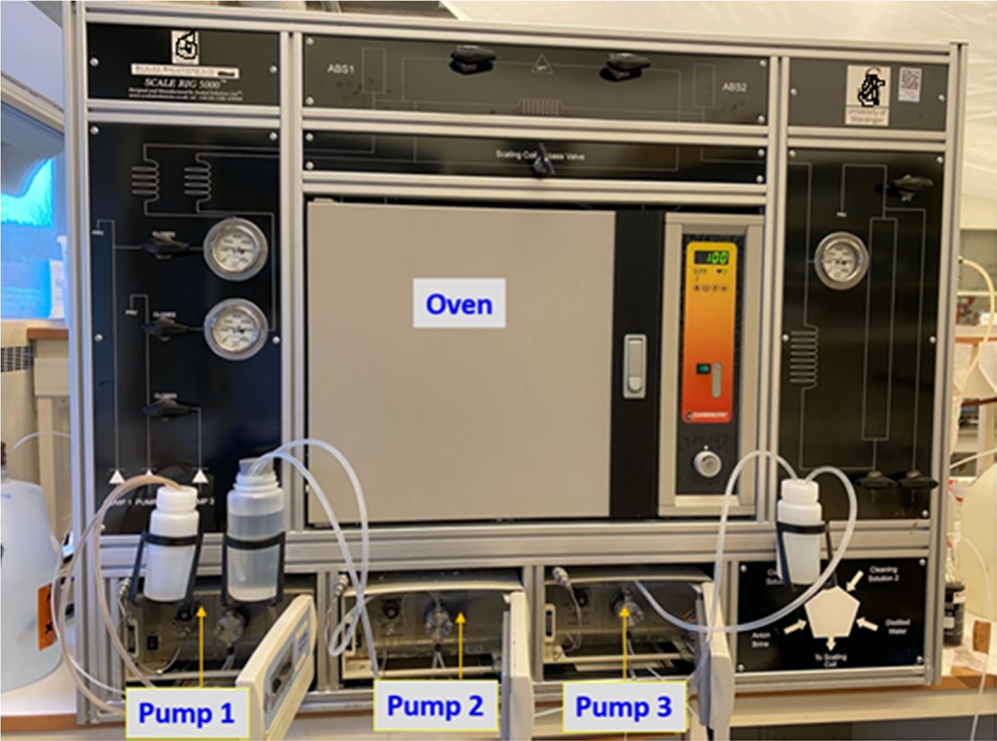
Picture of the dynamic tube-blocking scale rig used in this study.

An automated scale rig manufactured by Scaled Solutions Ltd., Scotland (Figure 2), was used to test and evaluate the inhibition performance of the commercial and the synthesized phosphonated products. The equipment consists of three pumps that flow aqueous solutions at the desired flow rate (10 mL/min) through a microbore 316 stainless steel coil (3 m long and 1 mm of internal diameter) placed inside an oven. The dynamic scale rig can tolerate quite high temperature and pressure manipulations (up to 300 bar and 200 °C), but for the present study, the parameters were set to 80 bar and 100 °C, respectively (ref).

**Figure 3:**
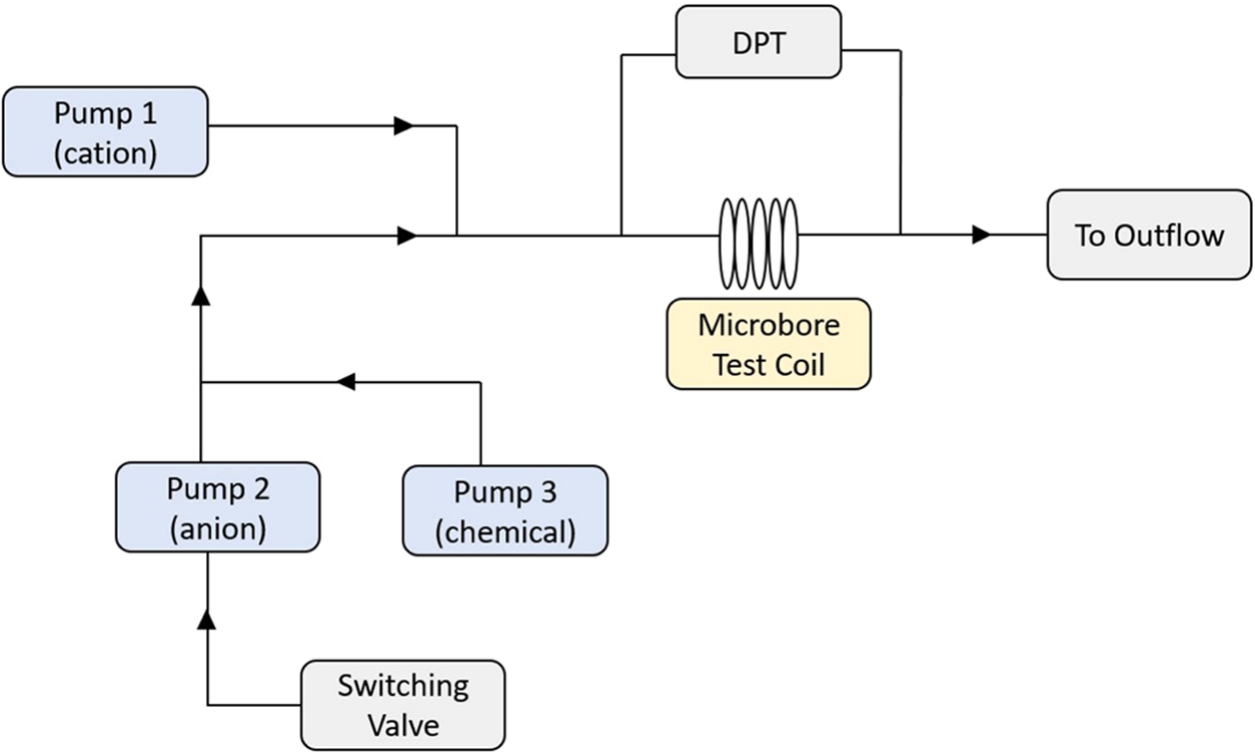
Schematic diagram of the dynamic tube-blocking scale rig.

The schematic diagram of the scale rig (Figure 3) helps to explain a typical test situation where each pump is responsible for pumping certain solutions through the test coil. Pumps 1 and 2 inject aqueous solutions of cations and anions, respectively, while the inhibitor solution is pumped by pump 3. The cleaning solutions, basic EDTA solution, and distilled water are also pumped by pump 2.

The automated scale rig is programmed to run three successive tests in each test run:

- The first test is “Blank,” where only the cation and anion brines are pumped until we have scale formation.
- The second test is “Chemical” and consists of several automated runs, each one lasting 1 h with inhibitor concentrations decreasing gradually at each stage, until at a certain concentration the tube blocking occurs.
- The third one is “Repeat Chemical,” which starts from the concentration one stage earlier than the step leading to rapid scale formation.

The stock inhibitor solution has an initial concentration of 1000 ppm with a pH range of ≈ 4–6 to ensure that when diluted during the test, there is negligible effect on the pH from the inhibitor solution. During the test, the brine and the inhibitor solutions are mixed to obtain a certain inhibitor concentration inside the test coil. A typical scale test starts from an inhibitor concentration of 100 ppm, with concentration gradually descending in each hour (50, 20, 10, 5, 2, and 1 ppm) until the scale formation occurs and the cleaning cycle begins. The concentration where rapid tube blocking happens is termed the fail inhibitor concentration (FIC) of the scale inhibitor. This is signaled by a sudden increase in the differential pressure (about 10 psi from the baseline) across the test coil. The test provides an assessment of the MIC value (minimum inhibitor concentration required to prevent scale formation). We consider a chemical with an FIC value within the range of 2–10 ppm to be performing excellent to fairly well toward scale inhibition under the test conditions described.

The synthetic brines used in this study are based on water produced in the Heidrun oilfield, Norway. Brines for the calcite scale are made using only formation water, while for barite scaling, a 50/50 mixture of formation water and the simulated seawater is used. The salt compositions are given in Table 1 below.

**Table 1:** Salt Composition for Brines 1 and 2 for Calcite and Barite Scaling

		calcite scaling			barite scaling		
ions	salts to be used	conc. (ppm)	brine 1 (g/3 L)	brine 2 (g/3 L)	conc. (ppm)	brine 1 (g/3 L)	brine 2 (g/3 L)
Na^+^	NaCl	39,020	148.77	148.77	19,510	115.92	105.12
Ca^2+^	CaCl_2._2H_2_O	2040	22.45		2040	15.93	
Mg^2+^	MgCl_2_.6H_2_O	530	13.30		530	40.98	
K^+^	KCl	1090	6.23		1090	5.76	
Ba^2+^	BaCl_2_.2H_2_O	570	3.04		570	1.53	
Sr^2+^	SrCl_2_.6H_2_O	290	2.65		290	1.32	
HCO_3_^–^	NaHCO_3_	1000		8.26			
SO_4_^2–^	Na_2_SO_4_				2960		13.14

### 2.5 Thermal Stability Tests

Thermal aging is particularly useful to find out whether the synthesized SIs can maintain their inhibition performance when tested for high-temperature reservoir applications, including squeeze treatments. For this test, a 5 wt % solution of the phosphonated derivatives was prepared in distilled water which was placed inside a sealed hard-glass tube fitted with a Teflon stopcock. The solution in the tube was subjected to three repetitive cycles of vacuum-refill (with nitrogen) before finally sealing off under a nitrogen atmosphere. The tube was then heated at 130 °C and maintained at that temperature for 7 days. Afterward, aliquots of the solution were diluted to 1000 ppm aqueous solutions to be tested in the scale rig. In general, we compare the aged products to the nonaged ones to determine their stability (e.g., maintain or lose inhibition performance) when exposed to high-temperature conditions.

### 2.6 Calcium Compatibility Tests

High concentrations of divalent cations, especially calcium ions, present in the formation water can cause serious problems if the scale inhibitor itself turns out to be incompatible with brines. The scale inhibitors can form an insoluble Ca–SI complex when mixed with formation brines, thereby precipitating out from the solution and can cause serious formation damage by blocking the pores of the formation rocks when used in squeeze treatments. Such precipitation of SI–Ca complexes is known for some aminomethylene phosphonate-based SIs. Therefore, it was imperative to check for the Ca-ion concentration tolerance level of the synthesized phosphonated SIs.

For the compatibility study, varying concentrations of calcium ions and SIs were mixed in synthetic brine, and the mixture was heated to check for the appearance of any insoluble particles over a span of 24 h. Four different dosages of SIs (100, 1000, 10,000, and 50,000 ppm) were mixed, respectively, with three different calcium ion concentrations (100, 1000, and 10,000 ppm) taken in 50 mL Duran glass bottles with plastic lids containing synthetic brine (NaCl 30,000 ppm) to obtain a total number of 12 glass bottles. Next, the bottles were shaken to be homogeneous, pH adjusted (in the range of 4.0–4.5), and kept in an oven at 80 °C for 24 h. The mixtures were observed after regular intervals of 30 min, 1, 4, and 24 h to check for any appearance of turbidity/precipitation due to the formation of insoluble Ca–SI complexes.

### 2.7 Seawater Biodegradability Tests

The biodegradability test determines whether a chemical will be safe to use when discharged offshore. Chemicals that are readily biodegradable pose no threat to their long-term exposure to the environment. There are specified internationally accepted standard methods (ISO, OECD) and regulations (GLP, ISO 9000) related to biodegradability tests. In general, the biodegradation test deals with a biochemical process that occurs when certain chemicals are consumed by microorganisms, and we get an estimate of oxygen consumption during the process. For testing the seawater biodegradability of the synthesized SIs, we followed the OECD 306 method. Details of this procedure have already been mentioned in earlier works from our lab.^19^ Here, an OxiTop Control manometric system (WTW, Germany) was used to measure biological oxygen demand (BOD). The OECD 306 test lasted for 28 days, and the flasks used in the test contained a mixture of seawater, nutrients, and the test chemical solution (1 wt % in water). For the test, three types of control flasks were prepared—(1) blank containing only seawater and nutrients served as baseline, (2) negative controls containing nutrients, autoclaved seawater, and the test chemical with a final concentration of 69 mg/L, and (3) positive controls with seawater, nutrients, and sodium benzoate (readily biodegradable substrate) at 100 mg/L. The percent biodegradability was calculated by comparing the BOD value and the calculated theoretical oxygen demand (ThOD). For the test, the collected seawater was kept in a dark room at 20 °C overnight before being transferred to 510 mL amber bottles the next day. The OxiTop was made ready before different nutrients were added to the amber bottles, along with measuring heads. Subsequently, the bottles were incubated for 3 h at 20 °C. After incubation, 1.8 mL of a 1% (w/w) solution of each test chemical was added to the test and to the negative control flask. The positive control flask contained 1.0 mL of a 30 g/L sodium benzoate solution. Next, the amber bottles were capped with measuring heads containing NaOH pellets to remove CO_2_. The bottles were placed on magnetic stirrers in an incubation cabinet. Data collection was begun instantly. Oxygen consumption was recorded over the 28 days of testing. Thereafter, the data was downloaded to the bottle heads and the ThOD was calculated for the SI before being classified in the OECD guidelines. Complete nitrification was accounted for. Before determining the percent biodegradability, blank oxygen values (BOD values representing background respiration in seawater) were subtracted from the BOD of each test.

## 3 Results and Discussion

### 3.1 Synthesis and Chemistry

The phosphonation of tetrasodium iminodisuccinate salt (TSIDS) to form TSIDS-P was performed via the Moedritzer–Irani reaction (Figure 1).^25^ The optimal condition for the synthesis was found to be 1.2 mol equiv of H_3_PO_3_, a total of 6 mol equiv of HCl (i.e., 2 equiv for one N atom), and 1.5 mol equiv of HCHO per one mole of the starting material. Attempts were made using a lower mole equivalent of acid, which resulted in almost no conversion of the starting material; also, we got almost no improvement in the performance during the scale test of the product. A small excess of HCHO and H_3_PO_3_ were used to ensure full conversion of the starting material. A reaction time of 24 h was found to give complete consumption of the starting material (as indicated by ^1^H and ^31^P NMR). This was further verified by extending the reaction time beyond 24 h, which did not give any change in the scale inhibition efficiency of the product obtained.

^1^H NMR spectra of the product showed a clear doublet at δ = 3.29 ppm, typical of the CH_2_ protons from the methylenephosphonate group (N–CH_2_–PO_3_H_2_). Further, in the ^31^P NMR spectrum, the presence of a characteristic triplet peak at δ = 6.29 ppm was indicative of the phosphonic acid group. The upfield shift of the peaks can be explained by the strong electron-withdrawing nature of four carboxylic groups (−COOH) in the vicinity.

Usually, after completion, the reaction was cooled to room temperature before it was neutralized and made ready for further testing. We have sometimes observed that upon cooling, a very small amount of crystals separated from the reaction mixture, making us wonder about the crystalized/precipitated materials. Those crystals were filtered out and washed with distilled water, and NMR spectra were recorded. Surprisingly, we did not see any such characteristic peaks of methylenephosphonate both in the ^1^H or ^31^P spectra, nor did it appear in the starting material. However, the whole solution, when neutralized after the reaction and checked for NMR, gave us enough proof of phosphonation, as mentioned above.

The phosphonated diethyleneaminosuccinate (TSEDAS-P) was also synthesized in a similar method as for TSIDS. We used a total of 7 mol equiv of HCl and 3 mol equiv of H_3_PO_3_ and HCHO (1.5 equiv per N atom). We tried to reduce the mole equivalent of phosphorous acid, which resulted in relatively poor inhibition performance of the chemical in the scale rig. With 3 mole equiv of phosphorous acid, we got a clear indication of phosphonation from the ^31^P NMR spectra. However, the spectra revealed two triplet peaks (respectively at δ = 8.93 and δ = 8.32 ppm) of roughly equal intensity, indicating a mixture of two products, possibly a monophosphonated and a bisphosphonated product, although the peaks seem quite close for this to be true.^26^^1^H NMR spectra of the TSEDAS-P were inconclusive in terms of assigning the methylphosphonate protons. Fractional crystallization attempts to get one product turned out to be unsuccessful. Further, the use of excess phosphorous acid or extending the reaction time to improve the ratio of bisphosphonate to monophosphonate were not advantageous as we obtained similar NMR spectra and the scale test results were also similar. Incomplete phosphonation of amines has been reported previously.^27^ Therefore, the mixed TSEDAS-P product was used in the following scale-related tests.

### 3.2 High-Pressure Dynamic Tube-Blocking Rig Test Results

The newly synthesized phosphonated chemicals and their respective unphosphonated starting materials were tested for their inhibition efficiencies toward the calcite and barite scale. They have also been compared with the classic phosphonated SIs ATMP and DETPMP. Tables 2 and 3 list FIC values for all of the chemicals tested in the high-pressure tube-blocking rig.

**Table 2:** Scale Inhibition Tube-Blocking Test Results for the Calcite Scale^a^

	1st blank	1st scale test		2nd scale test	
chemical	time (min)	conc. (ppm)	time (min)	conc. (ppm)	time (min.)
ATMP	11	20	44	20	46
DTPMP	10	10	20	10	22
TSIDS	9	50	11	50	11
TSEDAS	10	100	23	100	24
TSIDS-P	12	10	4	10	20
TSEDAS-P	11	5	23	5	28
TSIDS-P*	10	20	5	20	14
TSEDAS-P*	9	20	6	20	8

a Thermally aged products are marked with an asterisk *.

**Table 3:** Scale Inhibition Tube-Blocking Test Results for the Barite Scale

	1st blank	1st scale test		2nd scale test	
chemical	time (min)	conc. (ppm)	time (min)	conc. (ppm)	time (min)
ATMP	6	10	10		
DTPMP	6	10	13	10	12
TSIDS-P	7	100	20	100	13
TSEDAS-P	7	100	9	100	31

**Figure 4:**
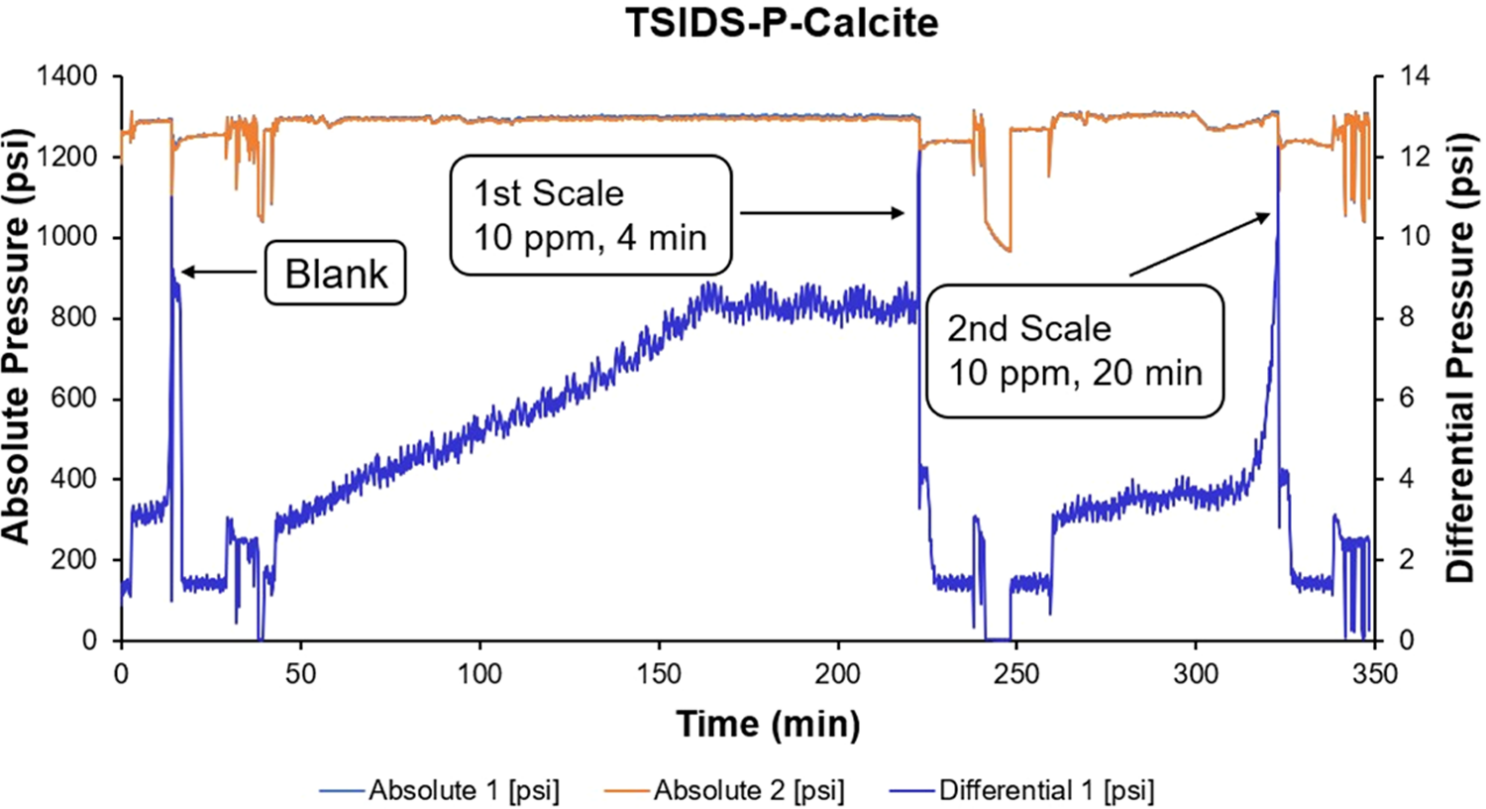
Schematic diagram of the dynamic test for TSIDS-P against calcite scaling.

For calcite scaling, both new phosphonated chemicals, TSIDS-P and TSEDAS-P, performed much better than the respective unphosphonated chemicals, TSIDS and TSEDAS. This shows the power of introducing as little as one aminomethylene phosphonate group into the chemical structure. TSIDS-P showed good calcite scale inhibition, with corresponding FIC values of 10 ppm after 4 and 15 min (Figure 4). This was a little better than the commercial product ATMP and a little worse than DTPMP. However, TSIDS-P exhibited a calcium incompatibility issue, as evident from the pressure vs. time graph in Figure 4. At the higher inhibitor concentrations of 100 ppm (ca. 50–110 min on the time axis) and 50 ppm (110–170 min), we see a gradual, steady increase of differential pressure. This is attributed to the formation and deposition of an insoluble Ca–SI complex.

**Figure 5:**
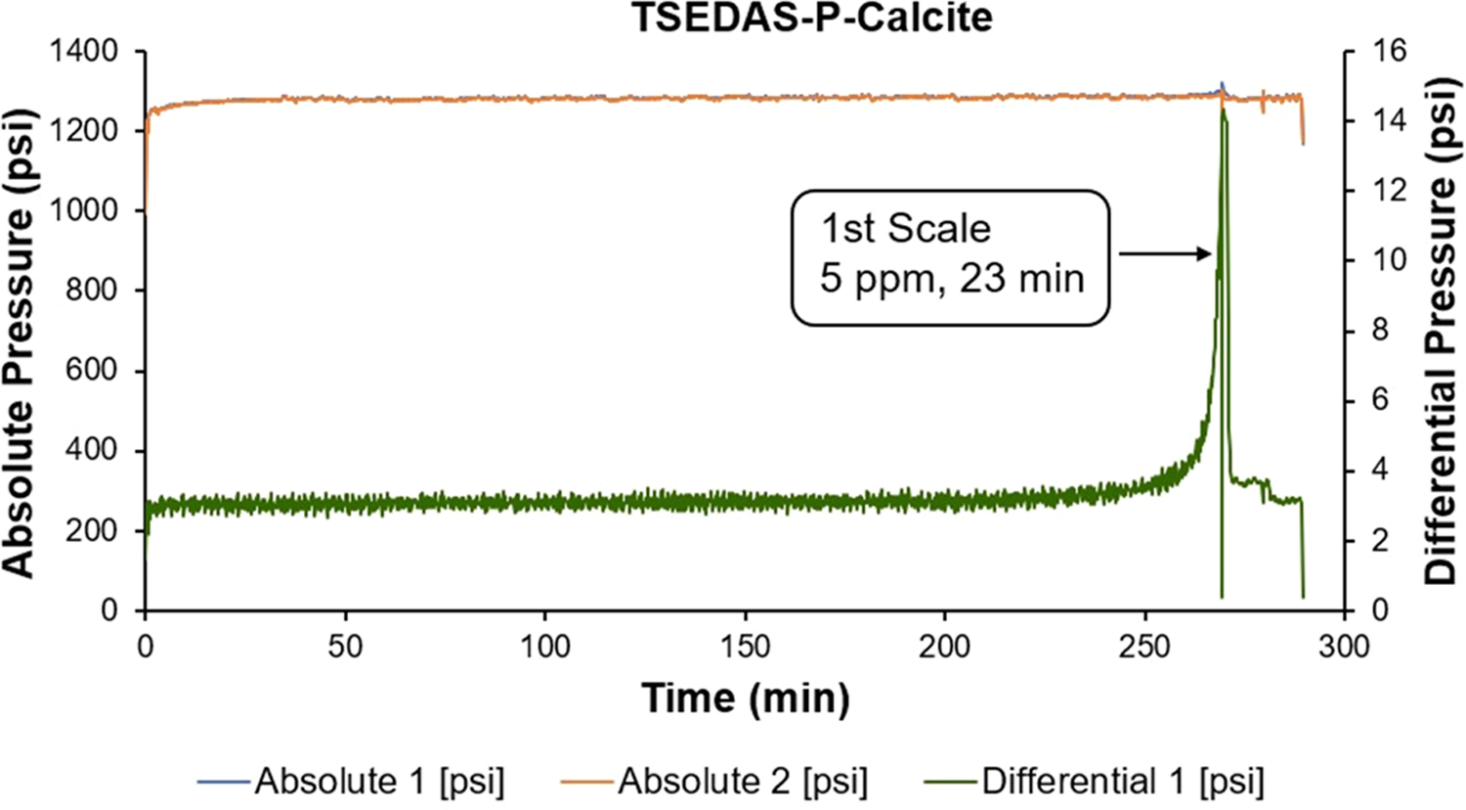
Schematic diagram of the dynamic test for TSEDAS-P against calcite scaling.

The phosphonated diamine analogue, TSEDAS-P, exhibited excellent calcite scale inhibition efficiency—the FIC value decreased to 5 ppm, better than TSIDS-P and the commercial phosphonates. The presence of bisphosphonated functional groups is probably the reason for the improved performance relative to TSIDS-P. Due to a technical error of the rig, we had to test the chemical in two separate runs instead of one full run (blank + chemical + repeat chemical). However, in both cases, we ended up at FIC = 5 ppm with almost identical run times (23 and 28 min). One of the runs is shown in Figure 5. In both runs with TSEDAS-P, we did not observe any calcium incompatibility; the graph remained mostly flat until rapid tube blocking due to calcite formation occurred. This essentially hints toward better calcium tolerance of TSEDAS-P compared to TSIDS-P. Separate calcium compatibility test results for both products are explored in more detail in Section 3.3 below.

Both of the new phosphonated chemicals, TSIDS-P and TSEDAS-P, were tested again after thermal aging at 130 °C for 7 days in the scale rig to determine whether the chemicals were degraded by being exposed to high temperatures. Reduced performance in the scale rig (i.e., if FIC shifted to a higher inhibitor concentration) would essentially indicate that the chemicals were not stable under a high temperature and thereby unsuitable for squeeze treatment at that temperature. Surprisingly, both TSIDS-P* and TSEDAS-P* were found to exhibit poorer performance toward calcite scale inhibition after thermal aging, although still better than the parent unphosphonated starting materials (Table 2). Thus, these chemicals are best to be used for low-temperature downhole applications or topside use where the temperature and duration in the system of the SI will be both lower.

The barite scale inhibition tests are summarized in Table 3. TSIDS-P performed rather poorly—corresponding FIC values for the barite scale were found to be only 100 ppm, with a runtime of 20 and 13 min. We obtained similar values for TSEDAS-P. The FIC was at 100 ppm with a runtime of only 10 and 31 min. Compared to the new chemicals, ATMP and DTPMP both gave a good performance with FIC values in the 10 ppm window.

This poor barite scale inhibition of the synthesized chemicals is probably due to the presence of only one methylenephosphonate group per N atom, which seems to be insufficient for good binding to barite nuclei and crystal surfaces. In contrast, ATMP has three methylenephosphonate groups on the central N atom and DTPMP has two such groups on the outer N atoms.

**Figure 6:**
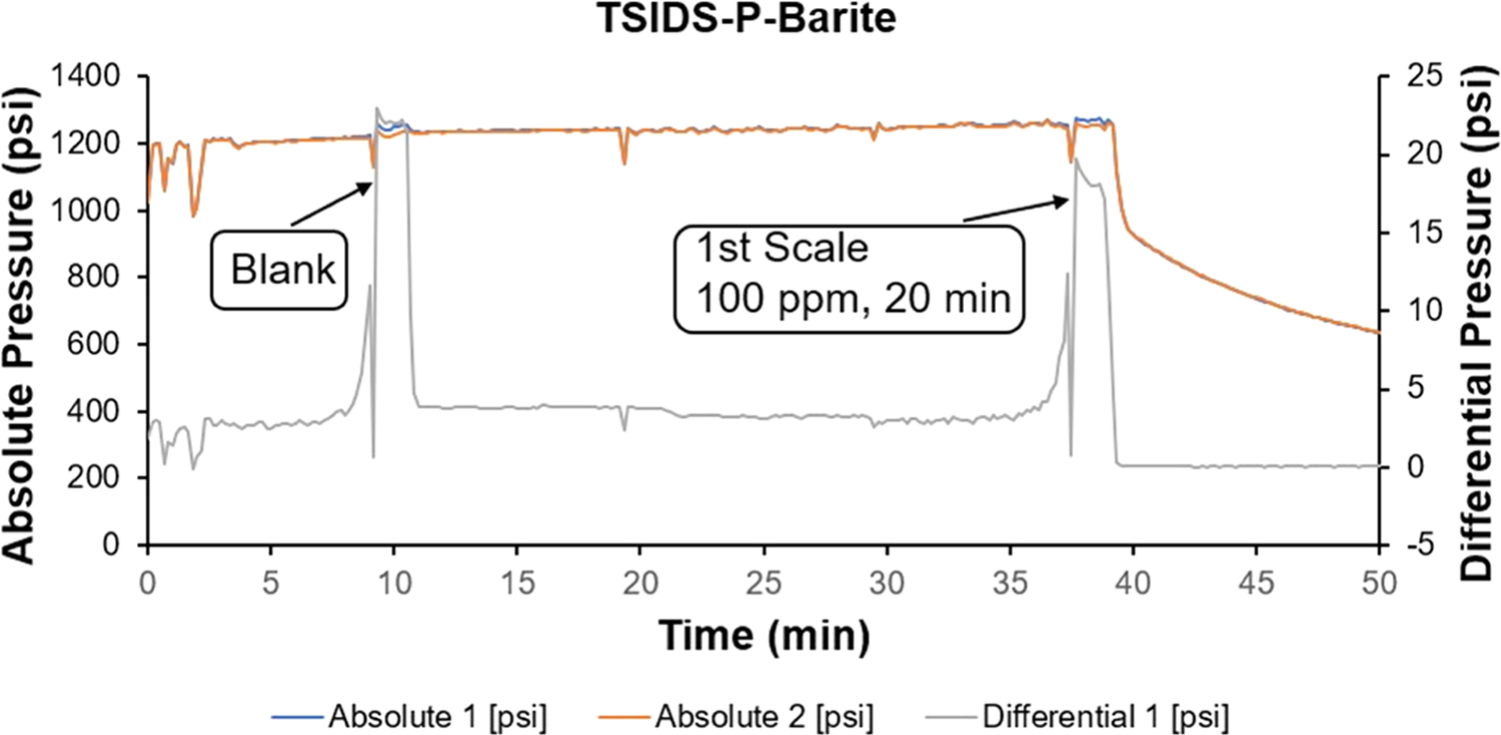
Schematic diagram of the dynamic test for TSIDS-P against barite scaling.

Since we already observed poor barite scale inhibition for both the phosphonated chemicals, we decided not to test these chemicals again after thermal aging (Figure 6).

### 3.3 Calcium Compatibility Test Results

As mentioned earlier in the Experimental Section, the calcium ion tolerance test is important to carry out, especially for aminomethylene phosphonate-based SIs, which tend to form insoluble Ca–SI complexes with medium to high concentrations of calcium ions in the formation water. The calcite scale inhibition tests had already shown some calcium incompatibility for TSIDS-P, which was why a more comprehensive set of compatibility jar tests were carried out.

Table 4 shows the results from the compatibility tests for TSIDS-P and TSEDAS-P with different inhibitors and Ca^2+^ ion concentrations. TSIDS-P with a high dosage of 50,000 ppm formed a precipitate with all three concentrations of Ca^2+^ (100, 1000, and 10,000 ppm) after 4 and 24 h. At lower dosages of TSIDS-P, there was no calcium incompatibility observed. As with the tube-blocking calcite test results, these results suggest that TDIDS-P is unlikely to be suitable for applications with raised temperatures, such as squeeze treatments, as the formation of Ca-TSIDS-P complexes could cause formation damage.

**Table 4:** Observations from Calcium Compatibility Experiments at 80 °C with Synthesized SIs in the Synthetic Brine (30,000 ppm NaCl)

		appearance for TSIDS-P				appearance for TSEDAS-P			
Ca^2+^ dose (ppm)	SI dose (ppm)	30 min	1 h	4 h	24 h	30 min	1 h	4 h	24 h
100	100	clear	clear	clear	clear	clear	clear	clear	clear
	1000	clear	clear	clear	clear	clear	clear	clear	clear
	10,000	clear	clear	clear	clear	clear	clear	clear	clear
	50,000	clear	clear	precipitate	precipitate	clear	clear	clear	clear
1000	100	clear	clear	clear	clear	clear	clear	clear	clear
	1000	clear	clear	clear	clear	clear	clear	clear	clear
	10,000	clear	clear	clear	clear	clear	clear	clear	clear
	50,000	clear	clear	precipitate	precipitate	clear	clear	clear	clear
10,000	100	clear	clear	clear	clear	clear	clear	clear	clear
	1000	clear	clear	clear	clear	clear	clear	clear	clear
	10,000	clear	clear	clear	clear	clear	clear	clear	clear
	50,000	clear	clear	precipitate	precipitate	clear	clear	opaque	opaque

In contrast, TSEDAS-P showed very good calcium compatibility. With TSEDAS-P, we did not observe any precipitate even after 24 h with high inhibitor concentration (50,000 ppm) and moderate Ca^2+^ concentration (1000 ppm). We observed only an “opaque/turbid” appearance and no precipitate (see Table 4) when tested with extreme Ca^2+^ and SI concentrations (10,000 and 50,000 ppm, respectively) after 4 and 24 h. Excellent Ca-compatibility was also observed in the dynamic test, where we did not experience any gradual increase in the pressure vs. time graph until rapid tube blocking took place. Thus, TSEDAS-P could be used for squeeze treatment injection even with high concentrations of Ca^2+^ present in the produced water.

### 3.4 Seawater Biodegradability Test Results

The OECD 306 test method was used to determine the biodegradability of both the phosphonated SIs and their starting material with a sodium benzoate solution as a control. Table 5 lists the results for all of the chemicals. The control gave a typical 28-day biodegradability (BOD28) value of 80.1% (average 3 tests). In comparison, the average BOD28 value of the iminosuccinate starting material TSIDS is 34.5%. This was an expected result as the chemical is already known as a readily biodegradable chelate by the OECD 301 test.^17^ The OECD 306 is generally a tougher biodegradation test as the levels of bacteria in seawater are much less than that in sewage-spiked freshwater.^2^ This explains why OECD 306 tests generally give lower BOD28 values than the OECD 301 tests.

**Table 5:** Biodegradability Test Results of SIs Using the OECD 306 Method after 28 Days

chemical	biodegradability, BOD28 (%)
Sodium Benzoate	79.5 (6 tests)
TSIDS	34.5 (6 tests)
TSIDS-P	72.6 (6 tests)
TSEDAS	0 (6 tests)
TSEDAS-P	2.7 (6 tests)

However, and to our surprise, the phosphonated analogue of TSIDS, TSIDS-P, was found to have outstanding biodegradability, with an average of 72.6% across all 6 bottles tested, placing it in the category of “readily biodegradable” in seawater. Originally, we carried out tests in triplicate. These first three bottles gave BOD28 values of 59.5, 68.1, and 80.0%. In the case of TSIDS-P, we were so surprised by the high biodegradation after 28 days that we initiated another 3 bottles using a new batch of seawater taken from the same location. These gave values of 71.9, 74.0, and 82.2%. Sodium benzoate as a control now gave an average of 78.8% in three tests, which was consistent with the first result and its known behavior. Thus overall, TSIDS-P gave an average BOD28 value of 72.6%.

According to OSPAR regulations, chemicals with >20% biodegradation can be discharged safely offshore Norway, but only if they meet other ecotoxicological requirements such as low toxicity (acute), low bioaccumulation, and low molecular weight. For chemicals with >60% biodegradation, the restrictions on the other requirements become easier since the chemical degrades so quickly and is therefore deemed to be less of a threat to the environment.

For TSIDS-P, with such a high biodegradation value, the chemical can be classed as readily biodegradable. Given the low bioaccumulation potential (high water-solubility), TSIDS-P could be a useful new green scale inhibitor for use in offshore regions with strict environmental requirements such as the North Sea.

In contrast, both TSEDAS and TSEDAS-P showed surprisingly poor biodegradability, with no positive degradation compared to the control bottles for TSEDAS and only 2.7% for TSEDAS-P. The tests were conducted at the same time and with the same batch of seawater as the tests with TSIDS and TSIDS-P. The BOD28 values were also confirmed in three further bottle tests later in the year on both chemicals. Looking at the related structures of TSIDS and TSEDAS and their phosphonate derivatives, these results can seem in conflict with each other, which was exactly why we repeated the biodegradation a second time. At this time, we have no good explanation for the significant difference in the BOD28 results for TSIDS-P and TSEDAS-P. We have conducted biodegradation tests using the same method and source of seawater for over ten years and the same personnel has been involved in the practical side of setting up the experiments. We intend to explore these results and related structural motifs in further biodegradation tests.

## 4 Conclusions

The test results obtained in this study are summarized in Table 6. Phosphonation of the iminosuccinates TSIDS, a biodegradable scale dissolver, and TSEDAS afforded two new phosphonated scale inhibitors. The phosphonate-iminodisuccinate TSIDS-P exhibited good scale inhibition against the calcite scale and good but limited Ca-compatibility but exceptional biodegradability. In the OCED306 seawater biodegradation test, TSIDS-P gave an average BOD28 value of 71.6%. TSIDS-P was not very thermally stable after anaerobic aging at 130 °C for 1 week, as judged by a worse calcite scale inhibition performance. The bisaminated chemical TSEDAS-P showed good calcite scale inhibition and excellent Ca-compatibility but poor biodegradability. Both inhibitors gave poor barite scale inhibition. TSIDS-P is therefore an excellent choice of green scale inhibitor for calcite scaling, particularly for the water treatment industry or treating topside applications (with moderate calcium concentrations) in the upstream oil and gas industry.

**Table 6:** Summary of Test Results for the Two New Phosphonated Iminodisuccinates

test	TSIDS-P	TSEDAS-P
calcite FIC	10 (4, 15 min)	5 ppm (23, 28 min)
barite FIC	100 ppm (13, 20 min)	100 ppm (9, 31 min)
thermal stability	<130 °C	not investigated
calcium compatibility	good	excellent
BOD28 (OECD 306)	72.6%	2.7%
